# Nonvalvular Papillary Fibroelastoma of the Left Ventricle: A Case Report and Review of the Literature

**DOI:** 10.1155/cric/2873563

**Published:** 2026-07-24

**Authors:** Aibek E. Mirrakhimov

**Affiliations:** ^1^ Department of Anesthesiology, Perioperative, Critical Care, and Pain Medicine, Division of Adult Cardiothoracic Anesthesiology, University of Kentucky, Lexington, Kentucky, USA, uky.edu

## Abstract

Papillary fibroelastoma (PFE) is a benign primary cardiac tumor that most commonly arises from valvular endocardium, whereas nonvalvular involvement, particularly within the left ventricle, is rare. Despite benign histology, PFEs are clinically relevant due to mobility and embolic risk. We report a case of a 64‐year‐old woman in whom a small nonvalvular left ventricular PFE was identified during evaluation for ventricular arrhythmias. Cardiac imaging demonstrated a mobile mass attached to the apical inferior left ventricular wall, which was successfully resected surgically under cardiopulmonary bypass with pathologic confirmation of PFE. We additionally performed a focused review of published cases of nonvalvular left ventricular PFEs, including lesions arising from the subvalvular mitral apparatus. The literature demonstrates heterogeneous clinical presentation, diverse anatomic attachment sites, and a strong preference for surgical management. This case and review highlight the importance of considering PFE in the differential diagnosis of left ventricular masses and support surgical excision for mobile nonvalvular lesions.

## 1. Introduction

Papillary fibroelastoma (PFE) is a relatively common benign primary cardiac tumor and represents the most frequent tumor of the cardiac valves [1]. In contrast, nonvalvular PFEs are distinctly uncommon and comprise a small minority of reported cases, with the left ventricle (LV) representing an especially rare site of origin [2–11]. Despite benign histopathology, PFEs are clinically significant because of their marked mobility and associated risk of systemic embolization, which may result in stroke, myocardial infarction, or sudden cardiac death.

The widespread use of advanced cardiac imaging modalities—particularly echocardiography and cardiac computed tomography (CT)—has increased incidental detection of small intracardiac masses; however, optimal management strategies for nonvalvular PFEs remain incompletely defined due to limited case‐level data. Surgical resection is generally favored in symptomatic patients or in the presence of high‐risk features. Here, we report a case of a nonvalvular LV PFE identified during evaluation for ventricular arrhythmias and successfully treated with surgical excision, and we review the existing literature on LV PFEs.

## 2. Case Report

A 64‐year‐old woman with hypertension and Type 2 diabetes mellitus underwent cardiac evaluation for symptomatic palpitations, frequent premature ventricular contractions, and episodic nonsustained ventricular tachycardia. Cardiac magnetic resonance (CMR) imaging and CT identified an approximately 7‐ to 1‐cm LV mass (see Figures 1 and 2). She had no history of stroke, transient ischemic attack, or peripheral embolic events at presentation.

**Figure 1:**
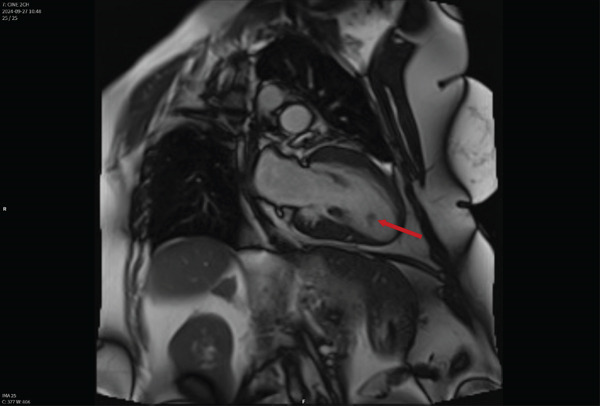
Cardiac magnetic resonance imaging, two‐chamber long‐axis view, demonstrating an approximately 1‐cm hypermobile abnormality (red arrow) in the mid‐to‐apical left ventricle.

**Figure 2:**
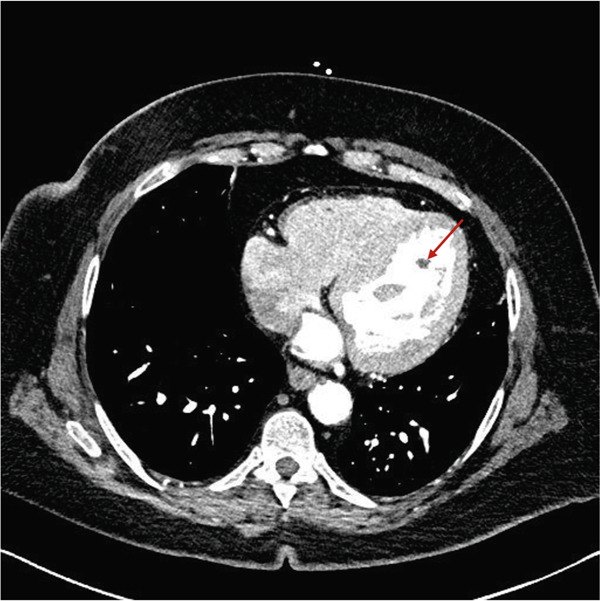
Axial cardiac computed tomography image demonstrating a small, 7‐mm spherical hypodense mass in the midleft ventricular cavity (red arrow).

The differential diagnosis included PFE, thrombus, vegetation, myxoma, Lambl excrescence, and primary or metastatic cardiac tumor. Thrombus was considered less likely because the lesion appeared discrete and pedunculated, without associated severe ventricular dysfunction or regional wall‐motion abnormality. Vegetation was also considered unlikely given the absence of fever, bacteremia, valvular involvement, or other clinical features of infective endocarditis. Given the lesion′s left‐sided location, mobility, and potential embolic risk, surgical excision was recommended after multidisciplinary discussion and shared decision‐making with the patient. A direct causal relationship between the mass and the patient′s ventricular arrhythmias could not be established.

After induction of general anesthesia and placement of invasive monitoring, intraoperative transesophageal echocardiography (TEE) confirmed a mobile LV mass measuring approximately 1 cm, attached by a stalk to the apical inferior wall, with otherwise normal cardiac findings. These findings are demonstrated in Videos S1 and S2 and Figure 3.

**Figure 3:**
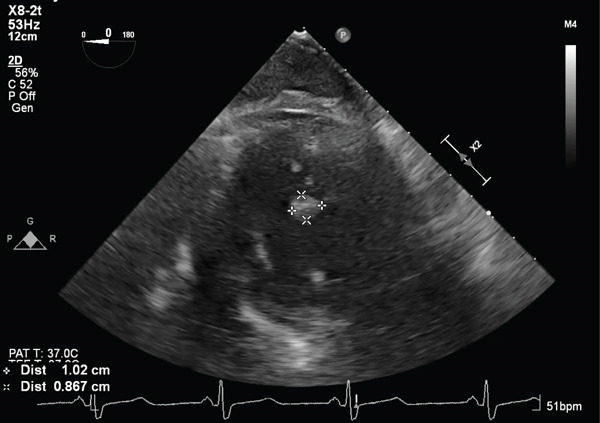
Frozen transgastric midpapillary TEE view demonstrating papillary fibroelastoma diameters by planimetry.

The patient underwent surgical excision under cardiopulmonary bypass. After aortic cross‐clamping and cardioplegic arrest, the mass was accessed through a ventriculotomy and completely excised. She was weaned from cardiopulmonary bypass without difficulty. Postexcision TEE demonstrated no residual mass and no apparent surgical complications.

Gross pathology examination demonstrated a yellow‐tan, friable soft tissue fragment measuring 1.1 × 1.1 × 0.4 cm. Microscopy showed classic features of PFE, including complex, arborescent paucicellular cores covered by endocardial lining cells. No atypia or thrombus formation was identified. One fragment contained endomyocardium, consistent with a possible attachment site, and no valvar tissue was present.

No perioperative embolic event was documented. During the postoperative course, the patient continued to have occasional premature ventricular contractions and runs of nonsustained ventricular tachycardia. Relevant postdischarge rhythm follow‐up was not available; therefore, whether tumor resection reduced the burden of premature ventricular contractions or nonsustained ventricular tachycardia cannot be determined from this case. Given the relative frequency of these arrhythmias in clinical practice, it is also possible that they were unrelated to the PFE. The patient was discharged to a rehabilitation facility on postoperative day 11.

## 3. Search Strategy

A focused English‐language literature review was performed to identify published cases of nonvalvular PFE involving the LV. PubMed/MEDLINE, Scopus, Google Scholar, and reference lists of relevant articles were searched from database inception through December 30, 2025. Search terms included combinations of “papillary fibroelastoma,” “cardiac papillary fibroelastoma,” “left ventricle,” “left ventricular,” “nonvalvular,” “left ventricular outflow tract,” “papillary muscle,” “chordae,” “mitral chordae,” and “subvalvular mitral apparatus.” Adult case reports, case series, and review articles were included if they described PFE arising from the LV cavity, LV wall, LV outflow tract, papillary muscles, or mitral chordal apparatus. Reports were excluded if lesions were confined to cardiac valves, involved only non‐LV sites, lacked sufficient anatomic detail, were unavailable in full text, or did not support a diagnosis of PFE. Duplicates were removed by comparing author names, publication year, patient characteristics, tumor location, and clinical presentation. Lesions involving the subvalvular mitral apparatus were included because the papillary muscles and chordae are integral LV structures and have similar diagnostic, embolic‐risk, and surgical implications.

## 4. Discussion

PFE is an uncommon benign primary cardiac tumor, accounting for approximately 5%–10% of all primary cardiac tumors and representing the most frequent tumor of the cardiac valves [1]. PFEs most commonly arise from valvular endocardium, particularly the aortic and mitral valves, whereas nonvalvular locations are distinctly uncommon [2–11]. Despite benign histopathology, PFEs are clinically significant because of their marked mobility and associated risk of systemic embolization, which may result in stroke, myocardial infarction, or sudden cardiac death.

The pathogenesis of PFE remains incompletely defined but appears more consistent with an acquired process than a true neoplasm. Proposed mechanisms include endothelial injury with subsequent microthrombus formation and abnormal organization of fibrin and elastic fibers in regions of elevated shear stress [1]. Histologically, PFEs are characterized by multiple avascular, frond‐like papillary projections composed of a dense central core of collagen and elastic tissue, surrounded by loose connective tissue and covered by endothelium. This distinctive architecture likely accounts for their marked mobility and embolic potential despite benign histology.

Although echocardiography remains the cornerstone of diagnostic imaging, CMR and cardiac CT may complement echocardiography in the evaluation of LV masses [12–15]. Cardiac CT provides high spatial resolution and can help define lesion location, attachment, and relationship to adjacent structures, whereas CMR provides tissue characterization that may help distinguish tumor from thrombus, myxoma, or vegetation. Thrombus typically lacks internal enhancement. Myxomas are usually larger, more heterogeneous, and often demonstrate variable enhancement related to vascularity, hemorrhage, calcification, or cystic degeneration. Vegetations are usually suspected in the appropriate clinical context, including fever, bacteremia, valvular destruction, or new regurgitation, and are typically attached to valve leaflets or prosthetic material rather than isolated LV endocardium. In contrast, PFE is typically small, highly mobile, pedunculated, and frond‐like, with variable late gadolinium enhancement related to fibroelastic tissue. CMR should therefore be viewed as adjunctive rather than definitive, particularly for small mobile lesions, for which histopathology remains the diagnostic standard.

Several registries and institutional series provide important context for the rarity of LV involvement. Klarich et al. [2] reviewed 54 patients with PFEs diagnosed by echocardiography with or without pathologic confirmation; only two tumors involved the subvalvular mitral apparatus (one papillary muscle and one chordae tendineae), nine arose in the left ventricular outflow tract (LVOT), and one was attached to the lateral LV wall. Sun et al. [3] reported 162 patients with pathologically confirmed PFE, including 13 with LV involvement, although specific attachment sites were not described. Kurup et al. [4] described 12 cases of so‐called “iatrogenic” PFEs occurring a mean of 18 years after a potential inciting exposure such as prior cardiac surgery or thoracic radiation, suggesting that endothelial injury may contribute to tumor development and, in some cases, multifocality. Within this cohort, three lesions were located in the LVOT, two were attached to the interventricular septum, and one was reported in an unspecified LV location.

In a pooled review of 725 cases, Gowda et al. [5] identified 100 nonvalvular PFEs, including 55 located within the LV without further anatomic specification. Strecker et al. [7] analyzed 84 surgically treated cardiac tumors; six were PFEs, of which two involved the LV without detailed localization. Kurmann et al. [8] evaluated longitudinal growth patterns in 279 patients with pathology‐proven PFE and included one LVOT lesion, demonstrating a mean annual growth rate of 0.5 ± 0.9 mm. Alozie et al. [9] reported a 27‐year surgical experience comprising 21 PFEs in 16 patients, with two tumors located in the LV, although specific attachment sites were not provided. More recently, Neupane et al. [10] performed a systematic review of 161 PFE cases associated with cerebrovascular events and identified LV involvement across several sites, including papillary muscle (*n* = 5), LVOT/interventricular septum/apex (*n* = 19), and chordal mitral structures (*n* = 2). Mrvic et al. [11] reported 12 PFEs over 16 years at a single institution, including one LV case without specified anatomic detail.

Collectively, these data confirm that LV PFEs are substantially less common than valvular PFEs. Moreover, LV involvement is often not the primary focus of large registries, resulting in incomplete reporting of key lesion characteristics such as precise attachment site, tumor size, mobility, and presenting symptoms. To address this gap, we performed a focused review of published reports of nonvalvular LV PFEs, including lesions arising from the subvalvular mitral apparatus, with findings summarized in Table 1 [16–102]. Of note, pathologic confirmation was not available in one reported case [42].

**Table 1:** Summary of nonvalvular PFE cases involving the left ventricle.

Reference	Year	First author	Country	Age and gender	Presenting symptoms	Location, size, and miscellaneous	Outcome
[16]	1989	Mazzucco A.	Italy	31‐year‐old male	Embolic coronary ischemic event.	1.6 cm adjacent to the anterolateral papillary muscle.	Surgical removal
[17]	1992	Uchida S.	Japan	62‐year‐old female	Mild exertional dyspnea.	LVOT. 2 cm in diameter. No evidence of LVOT obstruction.	Surgical removal
[18]	1993	Schuetz W.H.	Germany	57‐year‐old female	Embolic events.	Attached to interventricular septum. 1.5 cm in diameter.	Surgical removal
[19]	1995	Jobic Y.	France	69‐year‐old male	Dyspnea.	Inferoapical aspect of the LV. 1 cm in diameter	Surgical removal
[20]	1996	Ni Y.	Switzerland	69‐year‐old male	Incidental finding during bradycardia workup.	Apical anterior wall mass measuring 1.4 cm in diameter.	Surgical removal
[21]	1997	Espada R.	United States	62‐year‐old male	Embolic stroke.	3 × 2 cm mass between the anterolateral papillary muscle and the LV wall.	Surgical removal
[22]	1997	Tkebuchava T.	Switzerland	69‐year‐old male and 50‐year‐old male	Incidental findings during cardiac arrhythmia workup.	1.4‐cm mass in the anteroapical region of the LV in the 69‐year‐old and 1‐cm tumor attached to the chordae of the mitral valve.	Surgical removal
[23]	1999	Suzuki H.	Japan	54‐year‐old male	Incidentally found during workup of hyperthyroidism.	Mass attached to the mitral valve chordae. No information on the tumor size was provided.	Surgical removal
[24]	2000	Taniyasu N.	Japan	60‐year‐old female	Exertional dyspnea.	1.3 × 1 cm in the LVOT.	Surgical removal
[25]	2003	Eslami‐Varzaneh F.	United States	67‐year‐old female	Found during admission for heart failure. Comorbid aortic stenosis, mitral regurgitation and atrial fibrillation with history of remote embolic stroke.	2.0 × 1.5 × 1.2 cm mass in the LVOT. Also, the patient had additional tumors on the aortic and mitral valves.	Surgical removal
[26]	2003	Schoondyke J.W.	United States	71‐year‐old female	Found during workup of heart murmur.	~0.7‐cm mass on the lateral wall of the LV adjacent to the posteromedial papillary muscle.	Surgical removal
[27]	2003	Sato Y.	Japan	74‐year‐old male	Embolic cerebrovascular accident.	0.91 × 0.59 cm mass in the LVOT.	Surgical removal
[28]	2004	Tanaka H.	Japan	52‐year‐old male	Dyspnea. However, the patient had moderate combined mitral stenosis and regurgitation, moderate tricuspid regurgitation and atrial fibrillation.	Apical lateral wall; 7 mm in its maximum length. 4 years later, the patient presented with exertional dyspnea and was found to have moderate aortic insufficiency and two papillary fibroelastomas involving the aortic valve.	Surgical removal
[29]	2004	Irie Y.	Japan	59‐year‐old male	Embolic stroke.	A 5 × 6 cm mass was identified in the LVOT, with several adjacent polypoid lesions that were confirmed as papillary fibroelastomas on pathology.	Surgical excision
[30]	2005	Kim H.K.	South Korea	47‐year‐old male	Chest discomfort and palpitations.	2‐cm mass attached to the anterolateral wall of the LV.	Surgical excision
[31]	2005	Misumi T.	Japan	82‐year‐old female and 63‐year‐old male	Asymptomatic 82‐year‐old patient undergoing CABG and 63‐year‐old male with transient atrial fibrillation.	1.5‐cm mass attached to the anterolateral wall in an 82‐year‐old female and 2‐cm mass attached to the posterolateral wall in a 63‐year‐old male.	Surgical excision in both patients
[32]	2006	Kurian K.C.	United States	38‐year‐old female	Chest pain.	1.9 × 1.6 cm mass attached to the posteromedial papillary muscle.	Surgical excision
[33]	2006	Tamaru N.	Japan	66‐year‐old male	Embolic cerebrovascular accident.	0.8 × 1.4 cm mass attached to the lateral wall adjacent to the posteromedial papillary muscle of the LV.	Surgical excision
[34]	2006	Sá M.I.	Portugal	68‐year‐old male	Embolic cerebrovascular accident.	2 × 2 cm mass attached to the midlevel of the inferior LV wall.	Surgical excision
[35]	2006	Outomuro D.	Argentina	55‐year‐old male	Transient ischemic attack.	4.7 × 3.5 × 1.8 cm mass in the LVOT producing obstructive symptoms.	Surgical excision
[36]	2006	Kaneko Y.	Japan	81‐year‐old female	Discovered during palpitations workup.	1.5‐cm mass attached to the apical anteroseptal wall.	Surgical excision
[37]	2007	Company Campins M.M.	Spain	55‐year‐old male	Transient ischemic attack.	2 × 1.5 cm mass in the apex of LV.	Surgical excision
[38]	2007	Hino H.	Japan	45‐year‐old female and 68‐year‐old male	Discovered during routine workup in a 68‐year‐old male and incidentally discovered in a 45‐year‐old female during echocardiographic surveillance of rheumatic valvular disease.	0.7 × 0.9 cm mass in the LVOT in a 68‐year‐old male patient. No size or specific location within the LV cavity was not reported in a 45‐year‐old female patient.	Surgical excision
[39]	2008	Zurrú M.C.	Argentina	59‐year‐old female and 55‐year‐old male	Embolic cerebrovascular accident in both patients.	Mass attached to the lateral wall of the LV (no information on the size is provided) in a 59‐year‐old female and 4.7 × 3.5 × 1.8 cm in the LVOT with associated obstructive hemodynamics.	Surgical excision in both patients
[40]	2008	Je H.G.	South Korea	39‐year‐old male	Incidentally found during workup for hypertension and hypertrophic cardiomyopathy.	1.3 cm attached to the anterolateral papillary muscle.	Surgical excision
[41]	2008	Palecek T.	Czech Republic	46‐year‐old female	Incidentally found during workup for fever and arthralgias.	1.4 × 1.7 cm mass attached to the apical inferior wall of the LV.	Surgical excision
[42]	2008	Mutlu H.	United States	63‐year‐old male	Incidentally found during workup for dyspnea.	1 × 1 cm mass attached to the inferior wall of the LV. A 1 × 1 cm mass was attached to the inferior wall of the left ventricle. Although its characteristics were suggestive of papillary fibroelastoma, the patient did not undergo surgical excision or biopsy, so the diagnosis remains unconfirmed.	The patient declined surgery and was managed with warfarin anticoagula tion. No complications were reported over 4 years of follow‐up.
[43]	2008	Yamauchi S.	Japan	81‐year‐old male	The mass was incidentally discovered during evaluation for chest discomfort.	1‐cm mass in the LVOT.	Surgical excision
[44]	2008	Yuan S.M.	Israel	33‐year‐old male	The mass was incidentally discovered during the workup for pleuritic chest pain.	0.7‐cm mass attached to the chord of anterior mitral leaflet.	Surgical excision
[45]	2008	Sakaguchi H.	Japan	81‐year‐old male	Incidental finding.	2 × 1.5 cm mass in the LVOT.	Surgical excision
[46]	2009	Akita M.	Japan	72‐year‐old male	The mass was incidentally found during the workup for ischemic chest pain.	~1.5‐cm mass attached to the anterior papillary muscle.	Surgical excision
[47]	2009	Yoda M.	Japan	70‐year‐old male	Embolic cerebrovascular accident.	Imaging demonstrated a 1 × 0.8 cm mass within the LVOT, a second smaller adjacent mass, and multiple additional masses attached to the aortic valve.	Surgical excision
[48]	2009	Kobayashi Y.	Japan	68‐year‐old male	Incidentally found, asymptomatic.	Several masses were present within the LV cavity, the largest measuring 2.4 × 1.1 cm and attached to the LV septal wall.	Surgical excision
[49]	2009	Kumar T.K.	United States	41‐year‐old female	Found during workup of chest pain in the setting of Noonan syndrome and hypertrophic cardiomyopathy.	Multiple masses were present in both ventricles, with the largest LV mass measuring approximately 1 cm and attached to the anterolateral septum adjacent to the LVOT.	Surgical excision
[50]	2010	Alves R.J.	Brazil	71‐year‐old female	Discovered during evaluation for transient right upper extremity pain determined to be embolic in origin.	A 0.9 × 0.8 cm lesion was identified in the apical septal region of the LV. Histological examination of the arterial embolus confirmed papillary fibroelastoma.	The patient declined surgery, and 5‐year surveillance imaging demonstrated two masses (1.8 × 1.2 cm and 0.89 × 1.0 cm) adhered to the mid‐LV septal wall.
[51]	2010	Domenech A.	Argentina	59‐year‐old female	Embolic cerebrovascular accident.	1.12‐cm mass in the apical LV.	Surgical excision
[52]	2010	Park J.H.	South Korea	73‐year‐old female	Incidentally found.	1.7 × 1 cm mass attached to the inferior wall of the LV.	Surgical excision
[53]	2010	Shuto T.	Japan	80‐year‐old male	Incidentally identified during surveillance echocardiography following prior surgical aortic valve replacement.	0.7‐cm mass attached to the septal wall of the LVOT.	Surgical excision
[54]	2011	Haberka M.	Poland	Unknown	Unknown.	Several masses of unknown size were attached to the mitral valve chordae and located between the posteromedial papillary muscle and the lateral LV wall.	Unknown
[55]	2011	Mishra P.K.	United Kingdom	69‐year‐old male	Embolic cerebrovascular accident.	2.7 × 1.5 cm mass attached to the anterolateral papillary muscle.	Surgical excision
[56]	2011	Matijević V.	Croatia	41‐year‐old male	Embolic cerebrovascular accident.	1.5 × 0.9 cm mass attached to the posteromedial papillary muscle.	Surgical excision
[57]	2012	Samuels L.E.	United States	66‐year‐old male	Incidentally found during preoperative workup of right pulmonary mass.	1 cm in the LV apex.	Surgical excision
[58]	2012	Toeg H.D.	Canada	68‐year‐old male	Embolic cerebrovascular accident.	1.6 × 1.1 cm mass in the LV apex.	Surgical excision
[59]	2013	Hyun J.Y.	South Korea	65‐year‐old female	Incidentally found during chest pain workup.	1.8 × 1.7 cm mass attached to the apical inferior wall of the LV.	Surgical excision
[60]	2013	Murakami M.	Japan	85‐year‐old male	Incidentally found for atrial fibrillation surveillance.	1.8 × 2.5 cm mass attached to the mitral valve chord.	Surgical excision
[61]	2013	Mirdamadi A.	Iran	26‐year‐old male	Embolic cerebrovascular accident.	5 × 0.6 cm in the apical posterior wall. Additional mass was attached to mitral valve posterior leaflet.	Surgical excision
[62]	2013	Akagi H.	Japan	74‐year‐old male	Incidentally found during preoperative workup prior to orthopedic surgery.	1.9 × 1.5 cm mass attached to the apical inferior wall of the LV.	Surgical excision
[63]	2013	Kamdar F.	United States	66‐year‐old male	Embolic cerebrovascular accident.	0.9 × 0.9 cm mass attached to the septal wall of the LV.	Surgical excision
[64]	2014	Seto T.	Japan	75‐year‐old male	Incidentally found during chest pain workup.	1.4 × 1 cm mass attached to the LVOT.	Surgical excision
[65]	2014	Ariyoshi T.	Japan	81‐year‐old female	Incidentally found during workup prior to uterine surgery.	1.3‐cm mass in the LV apex.	Surgical excision
[66]	2014	Vallurupalli S.	United States	38‐year‐old male	Embolic cerebrovascular accident.	2‐cm mass in the apical lateral wall.	Surgical excision
[67]	2015	Kawamura I.	Japan	74‐year‐old male	Incidental finding.	2.1 × 2.1 cm mass attached to chordae of the mitral valve.	Surgical excision
[68]	2015	Diplaris K.	France	46‐year‐old male	Embolic cerebrovascular accident.	Small mass attached to the primary chordae of the mitral valve and smaller mass close to the anterolateral papillary muscle. Concomitant mass (0.9 × 0.9 cm) attached to the anterior leaflet of the mitral valve.	Surgical excision
[69]	2015	Kammerer I.	Germany	70‐year‐old male	Embolic cerebrovascular accident in 2005.	Tumor close to the posterior chordae of the mitral valve (specifics not provided). In 2014, the patient was incidentally found to have papillary fibroelastoma recurrence on the mitral valve.	Surgical excision
[70]	2015	Duke J.	Australia	53‐year‐old male	Discovered during workup of bacteremia.	1.9 × 1.5 cm attached to the apical lateral wall of the LV.	Surgical excision
[71]	2016	Antonopoulos A.S.	Greece	39‐year‐old male	Embolic events (popliteal artery, splenic, and suspected coronary artery).	5.0 and 1.8 cm attached to the anterolateral papillary muscle.	Surgical excision
[72]	2016	Ishida K.	Japan	78‐year‐old male	Incidental finding.	1‐cm mass attached to the septal wall of the LV.	Surgical excision
[73]	2017	Sharma R.	Canada	43‐year‐old female	Discovered during workup of coronary artery disease (of note, this patient had multiple risk factors including hypertension, diabetes mellitus, hyperlipidemia, and prior cardiac ischemia).	1.3 cm adjacent to the mid anterior wall.	Surgical excision
[74]	2017	Samanidis G.	Greece	73‐year‐old male	Incidental finding.	Mass attached to the posterior papillary muscle (no information on size was provided).	Surgical excision
[75]	2018	Page S.	Australia	72‐year‐old male	Discovered during workup of resistant hypertension.	1.3 × 1.3 cm in the apical septal wall of the LV.	Surgical excision
[76]	2018	Tanaka Y.	Japan	75‐year‐old male	Incidental finding.	1.5 × 1.2 cm mass attached to the LV apex between anterior and posterior papillary muscles.	Surgical excision
[77]	2018	Coats C.J.	United Kingdom	70‐year‐old male	Embolic cerebrovascular accident.	Mass in the LVOT (no information on size was provided).	Surgical excision
[78]	2018	Cianciulli T.F.	Argentina	69‐year‐old female	Discovered during workup for congestive heart failure (of note, this patient had concomitant severe mitral regurgitation and pulmonary hypertension).	2.19 × 1.46 cm mass attached to the posteromedial papillary muscle.	Surgical excision
[79]	2019	Popovic C.	Australia	56‐year‐old female	Embolic cerebrovascular accident.	Recurrence of multiple LV papillary fibroelastomas. The patient was reported to have papillary fibroelastomas resected in the past, though no specific location was provided.	Surgical excision
[80]	2019	Rigueira J.	Portugal	61‐year‐old female	Incidental.	1.4 × 1.4 cm in the mid‐LV.	Surgical excision
[81]	2020	de Klerk S.	United States	74‐year‐old male	Discovered during workup for dyspnea.	1.5 cm on the lateral wall just beneath the mitral valve annulus.	Surgical excision
[82]	2020	Stiru O.	Romania	74‐year‐old female	Discovered during workup for dyspnea and palpitations.	1.5 × 1.6 cm mass attached to the apical anteroseptal wall.	Surgical excision
[83]	2020	Ascaso M.	Spain	Not provided	Not provided.	Small mass in the LV apex.	Surgical excision
[84]	2021	Awad A.	Ireland	51‐year‐old female	Embolic cerebrovascular accident.	2.6 × 1.7 cm attached to the apical anteroseptal wall.	Surgical excision
[85]	2021	Nguyen G.T.	Vietnam	61‐year‐old female	Discovered during workup for ischemic heart disease.	1.4 × 1.8 cm mass attached to the anterolateral papillary muscle.	Surgical excision
[86]	2021	Sovic W.R.	United States	61‐year‐old female	Discovered during workup for palpitations.	0.8‐cm mass in the LV apex.	Surgical excision
[87]	2021	Im J.	South Korea	62‐year‐old male	Embolic cerebrovascular accident.	1.2 × 0.6 cm mass attached to the LV apex.	Surgical excision
[88]	2022	Galea N.	Italy	75‐year‐old female	Incidentally found. The patient had history of transient ischemic attacks.	1.5‐cm mass attached to the anterior papillary muscle.	Surgical excision
[89]	2022	Niwano A.	Japan	80‐year‐old male	Discovered during preoperative workup prior to parathyroidectomy.	1‐cm mass attached to the anterior papillary muscle.	Surgical excision
[90]	2022	Barbarossa A.	Italy	69‐year‐old male	Discovered during workup for history of multiple strokes.	1.3 × 0.9 cm mass in the LVOT.	Surgical excision
[91]	2023	Mitchell K.G.	United States	72‐year‐old female	Amaurosis fugax.	0.8‐cm mass attached to chordal apparatus of the mitral valve.	Surgical excision
[92]	2023	Balboa C.	Spain	66‐year‐old male	Embolic cerebrovascular accident.	1 × 0.8 cm mass at the mid anteroseptal wall.	Surgical excision
[93]	2023	Rocha B.M.L.	Portugal	81‐year‐old male	An incidental finding identified during surveillance in a patient with a history of bioprosthetic aortic valve replacement.	0.9 × 1.1 cm mass attached to the LV apex.	Surgical excision
[94]	2024	Pernigo M.	Italy	32‐year‐old female	Embolic finger ischemia.	0.8‐cm mass attached to the anterolateral papillary muscle.	Surgical excision
[95]	2024	Al Umairi R.S.	Oman	75‐year‐old male	An incidental finding identified during the evaluation of chest pain.	1 × 0.6 cm mass attached to the anterolateral papillary muscle.	Surgical excision
[96]	2024	Behjati M.	Iran	46‐year‐old female	Discovered during workup for vertigo and dyspnea.	2.35 × 1.96 × 1.65 cm mass attached to the chordae of the anterolateral papillary muscle.	Surgical excision
[97]	2024	Barron K.T.	United States	67‐year‐old female	Discovered during workup for dyspnea and palpitations.	1.3 × 1.1 cm mass attached to the LV apex.	Surgical excision
[98]	2025	Piber N.	Germany	73‐year‐old male	Incidental discovery.	1.2 × 0.7 cm mass attached to the lateral wall of LV.	Surgical excision
[99]	2025	Dziarmaga M.	Poland	55‐year‐old male	Discovered during evaluation for acute coronary syndrome.	1.8 × 0.9 cm mass near the LV apex.	Surgical excision
[100]	2025	Tada H.	Japan	79‐year‐old female	Incidental discovery.	1.7‐cm mass attached to the posterior papillary muscle.	Surgical excision
[101]	2025	Valencia‐Hernandez H.A.	Mexico	62‐year‐old female	Embolic cerebrovascular accident.	1.4 × 1.0 cm mass attached to the LV apex.	Surgical excision
[102]	2025	Masuda T.	Japan	74‐year‐old female	Discovered during evaluation for chest pain and palpitations.	1.5 × 1.5 cm mass attached to the LV apex.	Surgical excision

Across 91 reported patients included in Table 1, age was available for 89 and ranged from 26 to 85 years, with a mean age of 62.8 years (median: 66 years). Sex was reported for the same 89 patients, demonstrating a male predominance (62.9%) compared with female patients (37.1%). Age and sex were not provided in two reports [54, 83]. Our index patient—a 64‐year‐old woman—closely approximates the pooled mean age and aligns with the substantial minority of female presentations described in the literature.

For descriptive analysis, clinical presentation was assigned according to the primary trigger for imaging or diagnosis reported in each case. Embolic events included stroke, transient ischemic attack, coronary ischemia, peripheral arterial embolism, retinal ischemia, or other systemic embolic manifestations. Incidental cases included asymptomatic lesions or masses identified during unrelated evaluation, surveillance imaging, or preoperative assessment. When multiple symptoms were reported, the dominant presentation emphasized by the original report was used.

Incidental detection during evaluation for unrelated complaints, preoperative assessment, or surveillance imaging represented the most common reason for workup and diagnosis [17, 19, 22–26, 28, 30–32, 36, 38, 40–46, 48, 49, 52, 53, 57, 59, 60, 62, 64, 65, 67, 70, 72–76, 78, 80–82, 85, 86, 89, 93, 95–100]. The second most common clinical trigger for echocardiographic identification of LV PFE was an embolic or ischemic event, accounting for approximately one‐third of cases. These events included stroke or transient ischemic attack, coronary ischemia, limb or finger ischemia, and retinal ischemia [16, 18, 21, 27, 29, 33–35, 37, 39, 47, 50, 51, 55, 56, 58, 61, 63, 66, 68, 69, 71, 77, 79, 84, 87, 88, 90–92, 94, 101].

Symptom‐driven presentations without clear embolic syndromes included dyspnea or heart failure syndromes [17, 19, 24, 25, 28, 42, 78, 81, 82, 96, 97] and chest pain and discomfort syndromes [30, 32, 43, 44, 46, 49, 59, 64, 85, 95, 99, 102], whereas arrhythmia‐ or palpitation‐based evaluations were less frequent but clinically relevant [20, 22, 30, 31, 36, 82, 86, 97, 102]. Our case is notable in that the lesion was identified during evaluation for palpitations with frequent ventricular ectopy and nonsustained ventricular tachycardia, consistent with several reports in which PFEs were detected during rhythm evaluation rather than following embolic events [20, 30, 36, 86, 102]. A graphical summary of the clinical events preceding echocardiographic diagnosis is illustrated in Figure 4.

**Figure 4:**
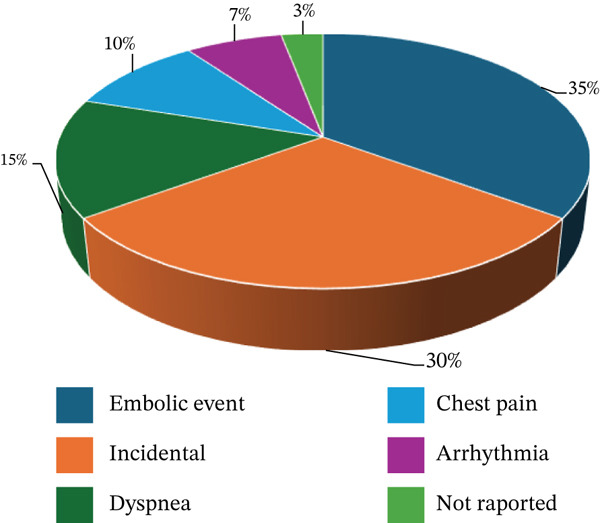
Clinical presentation leading to echocardiographic diagnosis among reported nonvalvular left ventricular papillary fibroelastoma cases (*n* = 91). Categories were assigned according to the primary clinical trigger for imaging or diagnosis. Embolic event: *n* = 32; incidental finding: *n* = 27; dyspnea/heart failure symptoms: *n* = 14; chest pain: *n* = 9; arrhythmia/palpitations: *n* = 6; not reported: *n* = 3.

Tumor location was categorized according to the reported anatomic attachment site. Lesions involving papillary muscles, chordae tendineae, or the mitral chordal apparatus were classified as subvalvular mitral apparatus. Lesions described within the LVOT or attached to the outflow septum were classified as LVOT. Lesions attached to the apical, septal, inferior, lateral, anterior, or other ventricular wall/cavity sites were classified as LV wall/cavity lesions.

A key feature of this review is the inclusion of PFEs attached to the subvalvular mitral valve apparatus, including papillary muscles and chordae tendineae, which represent a meaningful subset of reported nonvalvular LV PFEs. When lesions were categorized by reported attachment site, three principal anatomic patterns emerged: involvement of the subvalvular mitral apparatus, lesions arising from the LVOT, and tumors originating from the LV walls or cavity. The relative distribution of these anatomic attachment patterns is summarized in Figure 5.

**Figure 5:**
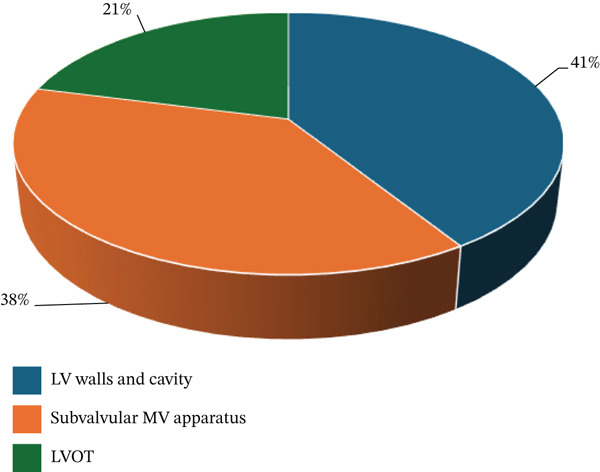
Anatomic distribution of reported nonvalvular left ventricular papillary fibroelastomas (*n* = 91). Tumor location was assigned according to the reported attachment site: left ventricular wall/cavity, *n* = 37; subvalvular mitral apparatus, *n* = 35; left ventricular outflow tract, *n* = 19. Subvalvular mitral apparatus included papillary muscle, chordal, and mitral chordal apparatus lesions. LVOT, left ventricular outflow tract.

Subvalvular mitral valve apparatus involvement was frequently described, with tumors attached to anterolateral, posteromedial, anterior, or posterior papillary muscles, as well as chordal structures [16, 21–23, 32, 33, 40, 44, 46, 54–56, 60, 67–69, 71, 74, 78, 85, 88, 89, 91, 94–96, 100]. Several reports lacked tumor size measurements despite describing chordal attachment [23, 68, 69], and one described multifocal involvement of the mitral valve complex without complete quantitative detail [54]. Clinically, this subvalvular distribution is relevant because lesions may be highly mobile and emboligenic and may require transmitral or transaortic surgical approaches depending on operative context.

PFEs involving the LVOT were also reported, sometimes with hemodynamic consequences when lesions were large or associated with adjacent tumors [17, 24, 25, 27, 29, 35, 38, 43, 45, 47, 53, 64, 77, 90]. Giant or bulky LVOT PFEs were described with obstructive physiology or multiple adjacent lesions [29, 35, 39]. Notably, some LVOT cases did not report tumor size [77]. Given proximity to the aortic valve and potential for outflow obstruction or embolization, this anatomic site may carry heightened clinical concern.

Many PFEs arose from the LV walls and cavity, including apical, inferior, lateral, anterior, septal, and anteroseptal regions [18–20, 28, 30, 34, 36, 37, 39, 41, 42, 48–55, 57–59, 65, 66, 70, 72, 73, 75, 82, 84, 87, 92, 97–99, 101, 102]. Our lesion originated from the apical inferior wall, mirroring the recurrent observation that the LV apex and adjacent wall segments are common nonvalvular sites. This distribution may reflect local flow conditions, shear stress, and endothelial microinjury as proposed mechanisms for PFE development, although definitive causality cannot be inferred from case‐level data.

Among cases reporting tumor dimensions, the maximum size ranged from approximately 0.7 to 6.0 cm, with many lesions clustering around 1–2 cm. Several reports lacked numeric size data, particularly for chordal or subvalvular lesions and recurrent tumors [23, 68, 69, 74, 77, 79, 83], and one report described multiple masses without measurement [54]. In our patient, the mass measured approximately 7 mm on cardiac CT and 1.0 cm on intraoperative TEE, placing it at the lower end of the reported size spectrum yet still clinically relevant given its mobility.

Management across published reports was overwhelmingly surgical, reflecting both perceived embolic risk and referral or publication bias. Nearly all patients underwent surgical excision, whereas medical management or observation was described in only two cases: One patient declined surgery and was managed with anticoagulation and surveillance without complications over 4 years [42], and another declined surgery despite embolic presentation, with subsequent imaging demonstrating persistent lesions [50]. One report did not provide management or outcome data [54]. Collectively, these observations suggest that when a mobile LV mass is suspected to represent PFE, particularly in the setting of embolic symptoms, surgical excision is commonly pursued.

Given the limited evidence base, management of suspected nonvalvular LV PFE should be individualized rather than protocolized. Figure 6 provides a practical diagnostic and therapeutic approach to a suspected nonvalvular LV mass. Initial evaluation should define mobility, attachment site, size, embolic or obstructive symptoms, arrhythmia context, and imaging features suggesting thrombus, vegetation, myxoma, or malignancy. Surgical excision is generally favored for left‐sided mobile or pedunculated lesions, symptomatic lesions, lesions associated with embolic events, or masses with uncertain diagnosis when operative risk is acceptable. Conservative management with surveillance imaging, with or without antiplatelet therapy, may be reasonable for selected small, immobile, asymptomatic lesions or patients with prohibitive surgical risk, although supporting evidence is limited.

**Figure 6:**
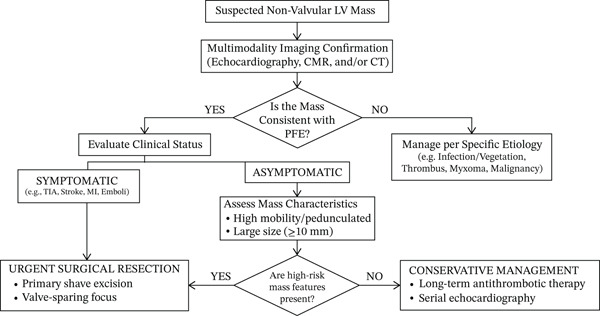
Clinical triage pathway for suspected nonvalvular left ventricular masses.

Our case aligns with this prevailing approach. The lesion was mobile, nonvalvular, and identified during arrhythmia evaluation, prompting surgical excision under cardiopulmonary bypass with intraoperative TEE confirmation of complete removal and no immediate complications. The operative strategy required ventriculotomy to access an apical inferior wall tumor—an approach that may be necessary for select apical or deep mural attachments but introduces theoretical risks such as ventricular dysfunction, arrhythmogenic substrate, or bleeding. Because published cases inconsistently report operative access routes and myocardial protection strategies, meaningful comparison of surgical techniques is not possible. Nonetheless, the predominance of surgical management in the literature likely supports resection as the dominant real‐world strategy for these lesions when mobility, symptoms, or embolic history are present.

In our patient, an apical ventriculotomy was used because the mass was attached to the apical inferior LV wall. This approach provides direct exposure for deep apical or mural lesions but may theoretically carry long‐term risks, including ventricular scar formation, regional wall‐motion abnormality, bleeding, or scar‐related ventricular arrhythmias. Alternative approaches, including transaortic, transmitral, thoracoscopic, or video‐assisted techniques, have been described for selected LV PFEs and may reduce the need for direct ventriculotomy when tumor location and exposure permit [54, 58, 79]. However, the optimal approach is individualized according to tumor attachment site, mobility, surgical exposure, and the need for complete excision.

Although this patient presented with premature ventricular contractions and nonsustained ventricular tachycardia, the arrhythmogenic role of LV PFE remains uncertain. Several reported LV PFEs have been discovered during evaluation for palpitations, ventricular ectopy, atrial arrhythmia, or chest pain with palpitations [18, 26, 32, 78, 82, 98]. These reports support a possible association, but not causality. Mechanistically, a highly mobile apical LV mass could theoretically produce repetitive mechanical contact, analogous to a “whiplash effect,” on adjacent endocardium, myocardium, or Purkinje‐rich subendocardial tissue. This concept is supported indirectly by mechanoelectric coupling literature showing that acute mechanical stimulation, local tissue deformation, and stretch‐activated currents can trigger ectopy [99, 100]. Purkinje fibers may also participate in mechanically induced ventricular arrhythmias. However, this mechanism has not been directly proven for PFE. In our case, ventricular ectopy and runs of nonsustained ventricular tachycardia persisted during hospitalization, and postdischarge rhythm monitoring was unavailable. Therefore, the mass may have been associated with arrhythmia‐driven discovery, but causality cannot be inferred from the current literature.

This review has important limitations. The evidence base consists predominantly of case reports and small case series, which are subject to publication bias, incomplete reporting, and preferential publication of surgically managed or clinically dramatic cases. Tumor mobility, size, attachment site, embolic history, operative approach, and follow‐up duration were inconsistently reported. As a result, pooled estimates from these reports should be interpreted descriptively rather than as true incidence, risk, or treatment‐effect estimates. Although surgical excision was the dominant management strategy among published cases, this likely reflects selection and reporting bias and should not be interpreted as definitive evidence that all nonvalvular LV PFEs require resection.

In summary, nonvalvular LV PFEs affect predominantly older adults with a male skew but occur across a wide adult age range, including younger patients with embolic stroke. Clinical presentation is heterogeneous, with embolic events and incidental detection accounting for most diagnoses, whereas arrhythmia‐driven discovery as in our case is less common but well described. Lesions arise from distinct anatomic clusters, including subvalvular mitral structures, the LVOT, and LV walls or apex, each with potential implications for surgical access. Although tumor size varies widely, even small mobile lesions may be clinically consequential, reinforcing why most reported cases proceeded to surgical excision.

## 5. Conclusion

Nonvalvular LV PFEs are rare but clinically relevant because mobility and left‐sided location may confer embolic risk. This case highlights the role of multimodality imaging and intraoperative TEE in diagnosis, surgical planning, and confirmation of complete resection. Published cases suggest heterogeneous presentation, frequent involvement of the subvalvular mitral apparatus or ventricular wall, and a strong reported preference for surgical excision. However, available evidence is derived mainly from case reports and small series, with substantial publication and selection bias. Management should therefore be individualized according to symptoms, mobility, attachment site, embolic history, operative risk, and patient preference.

## Funding

No funding was received for this manuscript.

## Conflicts of Interest

The author declares no conflicts of interest.

## Supporting Information

Additional supporting information can be found online in the Supporting Information section.

## Supporting information


**Supporting Information 1** Video S1. Transgastric two‐chamber TEE view demonstrating a mobile papillary fibroelastoma attached to the apical inferior left ventricular wall.

**Supporting Information 2** Video S2. Transgastric midpapillary TEE view demonstrating a mobile papillary fibroelastoma adjacent to the inferior left ventricular wall.

## Data Availability

Research data are not shared.
